# Virtual spherical-shaped multicellular platform for simulating the morphogenetic processes of spider-like body axis formation

**DOI:** 10.3389/fcell.2022.932814

**Published:** 2022-08-12

**Authors:** Motohiro Fujiwara, Yasuko Akiyama-Oda, Hiroki Oda

**Affiliations:** ^1^ Laboratory of Evolutionary Cell and Developmental Biology, JT Biohistory Research Hall, Takatsuki, Japan; ^2^ PRESTO, Japan Science and Technology Agency (JST), Kawaguchi, Japan; ^3^ Department of Microbiology and Infection Control, Faculty of Medicine, Osaka Medical and Pharmaceutical University, Takatsuki, Japan; ^4^ Department of Biological Science, Graduate School of Science, Osaka University, Toyonaka, Japan

**Keywords:** embryogenesis, body plan, arthropod, body axis formation, cell vertex model, mathematical modeling

## Abstract

Remodeling of multicellular architecture is a critical developmental process for shaping the axis of a bilaterally symmetric animal body and involves coordinated cell–cell interactions and cell rearrangement. In arthropods, the early embryonic process that leads to the segmented body axis varies at the cellular and molecular levels depending on the species. Developmental studies using insect and spider model species have provided specific examples of these diversified mechanisms that regulate axis formation and segmentation in arthropod embryos. However, there are few theoretical models for how diversity in the early embryonic process occurred during evolution, in part because of a limited computational infrastructure. We developed a virtual spherical-shaped multicellular platform to reproduce body axis-forming processes. Each virtual cell behaves according to the cell vertex model, with the computational program organized in a hierarchical order from cells and tissues to whole embryos. Using an initial set of two different mechanical states for cell differentiation and global directional signals that are linked to the planar polarity of each cell, the virtual cell assembly exhibited morphogenetic processes similar to those observed in spider embryos. We found that the development of an elongating body axis is achieved through implementation of an interactive cell polarity parameter associated with edge tension at the cell–cell adhesion interface, with no local control of the cell division rate and direction. We also showed that modifying the settings can cause variation in morphogenetic processes. This platform also can embed a gene network that generates waves of gene expression in a virtual dynamic multicellular field. This study provides a computational platform for testing the development and evolution of animal body patterns.

## 1 Introduction

Multicellular animals comprise more than 20 phyla, each with a different basic body plan (Brusca and Brusca, 2003; Valentine, 2004; Willmore, 2012). Body plan formation is achieved through cell proliferation and differentiation, cell movement and rearrangement, and cell–cell interaction and communication, which are controlled by the genome and cell mechanics (Forgacs and Newman, 2005). Genome information is inheritable but changeable over generations, with the body-forming process being able to diversify without disrupting the traits of the phylum (Richardson, 1995; Galis et al., 2002; Raff, 2012). How these modifications of the body-forming processes can occur during organism evolution is a fundamental question required for understanding the source of animal diversity.

**FIGURE 1:**
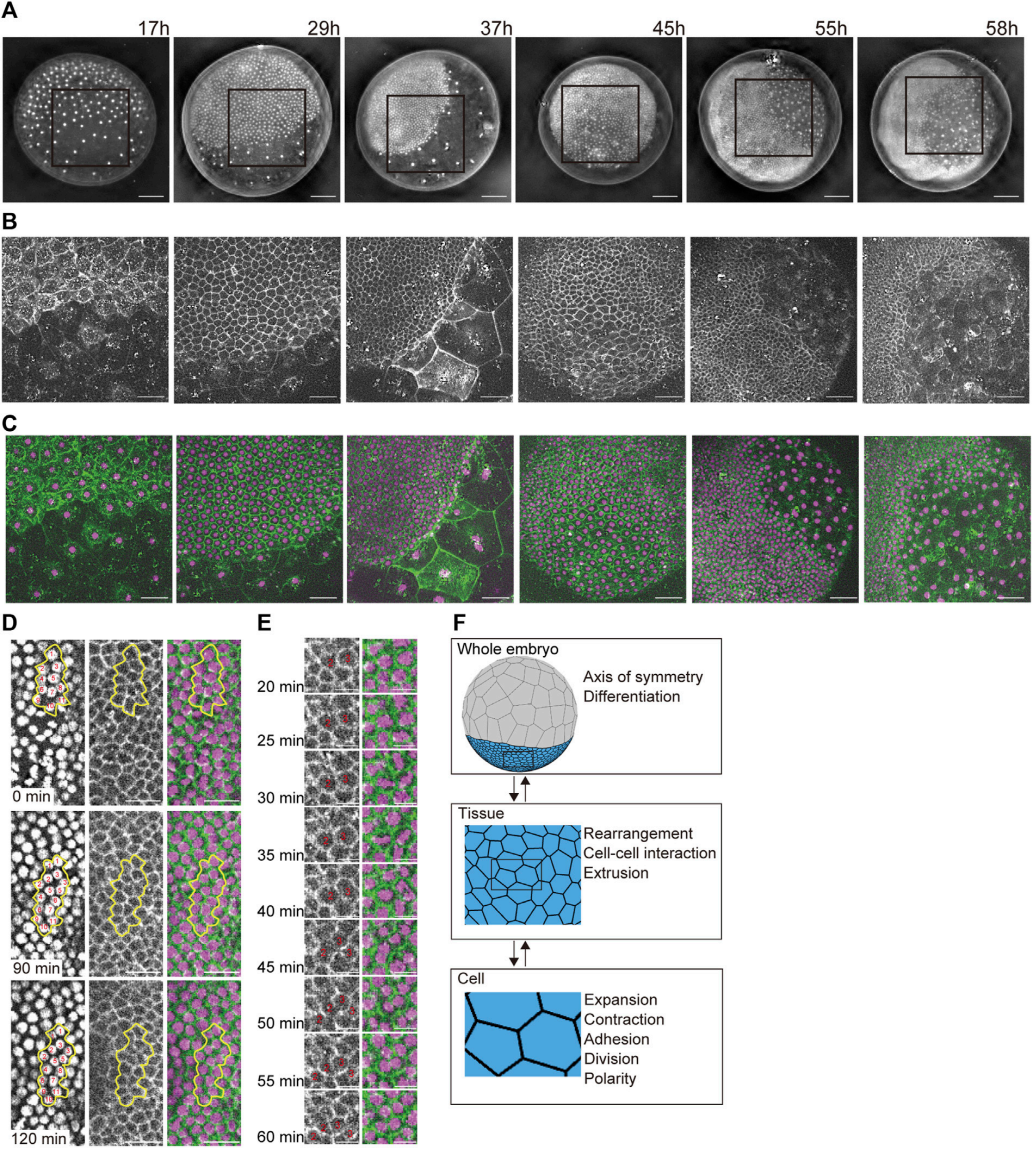
Developing multicellular architecture of the *P. tepidariorum* embryo. **(A–C)** Extended depth-of-field images of whole embryos at different developmental stages in which DNA [**(A,C)** in magenta] and F-actin [**(B,C)** in green] are labeled with vital fluorescent dyes. Time after egg laying (AEL) is indicated. For each embryo, the region boxed in **(A)** is magnified in **(B,C)**. **(D,E)** Time-lapse images of mediolaterally oriented cell intercalations and variously oriented cell divisions in the future thoracic region of the ectoderm in a live embryo labeled for DNA and F-actin. In **(D)**, individual cells grouped together outlined with yellow lines are numbered to aid tracking. In **(E)**, cell divisions are highlighted at higher temporal resolution. See Supplementary Movie S2 for details. Scale bars = 100 μm in **(A)**, 50 μm in **(B,C)**, 25 μm in **(D)**, and 10 μm in **(E)**. **(F)** Schematics of three hierarchical layers in our mathematical modeling of a spider embryo-like multicellular assembly and the phenomena/properties assigned to each layer. See Supplementary Movie S1 for details.

Early embryonic development in animals in the phylum Arthropoda is characterized by body axis formation and segmentation (Scholtz and Wolff, 2013). Cellular and molecular studies of a wide range of species, including the fruit fly *Drosophila melanogaster* (Irvine and Wieschaus, 1994), the red flour beetle *Tribolium castaneum* (Benton et al., 2013; Benton, 2018), the amphipod crustacean *Parhyale hawaiensis* (Sun and Patel, 2019), and the common house spider *Parasteatoda tepidariorum* (Oda and Akiyama-Oda, 2020), have revealed that the processes and mechanisms of body axis formation and segmentation vary substantially depending on the species despite conserved gene expression patterns during mid-embryogenesis (Liu and Kaufman, 2005; Peel et al., 2005; Sachs et al., 2015; Oda et al., 2020). The variation in early development among arthropods might be linked to the diversity in size, shape, composition, and other properties of the egg, which reproductive strategies associated with environmental adaptation (Scholtz and Wolff, 2013). Hence, species richness in phylum Arthropoda implies high flexibility and evolvability of its developmental systems (Stansbury and Moczek, 2013; Thomas et al., 2020). However, this evolutionary diversity is not easily testable in real organisms; therefore, mathematical modeling of arthropod embryos and simulation of their development contribute to investigating how early developmental processes are diversified. In many cases, tissue morphogenesis dynamics are accompanied by the development of gene expression patterns (Irvine and Wieschaus, 1994; Akiyama-Oda and Oda, 2010). Spatially periodic stripe formation associated with body-axis segmentation in arthropod and vertebrate embryos provides representative examples of these types of patterning processes in dynamic cellular fields. Studies of these examples have highlighted waves of gene expression that behave in various modes to generate periodic stripe patterns (Sarrazin et al., 2012; Hubaud and Pourquié, 2014; Akiyama-Oda and Oda, 2020). Because segmentation in the *Drosophila* blastoderm embryo occurs mostly in a syncytial environment, this popular model system only provides limited information about the relationship between pattern formation and tissue field dynamics. In contrast, similar to many other arthropod embryos, body axis formation and segmentation in the spider embryo occurs in the cellular field (Figure 1A; Kanayama et al., 2010; Hemmi et al., 2018; Akiyama-Oda and Oda, 2020).

An increasing number of mathematical modeling studies have simulated the dynamics of multicellular assemblies (Goriely, 2017), with many using cell vertex models in which each vertex follows motion equations based on cell mechanics (Honda, 1983; Farhadifar et al., 2007; Fletcher et al., 2014). Indeed, two-dimensional cell vertex models have effectively simulated the growth and morphogenesis of *Drosophila* epithelial tissues (Aliee et al., 2012; Kong et al., 2017). These models assume that the cortical actomyosin network and adherens junctions are localized at the apicolateral portions of cell–cell contacts (Fletcher et al., 2014). These adhesions play a major mechanical role in regulating cell size, shape, and behavior (Lecuit and Lenne, 2007; Paluch and Heisenberg, 2009). Actomyosin activity generates cortical tension on individual cells in a tissue, while adhesions resist tension and transform it into tissue-level tension (Heer and Martin, 2017). Cortical tension anisotropy is associated with planar cell polarity in epithelial tissues (Bertet et al., 2004; Keller, 2006). This tension can function as part of the mechanism of cell–cell intercalation to orient the movement of cell populations (Bertet et al., 2004; Blankenship et al., 2006), and differential tensions at the cell–cell interface can lead to local cell sorting (Landsberg et al., 2009).

The use of cell vertex models has been extended to three-dimensional (3D) tissue shaping (Honda et al., 2008; Alt et al., 2017; Okuda et al., 2018), which considers both the volume and 3D shape of individual cells. These developments in cell vertex models may complicate the handling parameters and increase the burden on calculations, which limits the number of cells which can be considered. Spider embryos undergoing segmentation along the emerging body axis are comprised of more than 3,000 cells (Akiyama-Oda and Oda, 2020), each of which has dynamic states of gene expression and dynamic interactions with surrounding cells. The simplicity of the modeling design is, therefore, key to reproducing the function of the animal embryo for morphogenesis and pattern formation.

Here, we propose a cell vertex model with a spherical surface in which a multicellular system deforms spatiotemporally based on cell dynamics. We attempted to model an arthropod-like whole embryo by mimicking the common house spider (*P. tepidariorum*) embryo. The proposed model helps us to understand the diversity of the body axis-forming processes. We also showed an expansion of the vertex model by introducing gene expression patterning based on molecular networks.

## 2 Materials and methods

### 2.1 Structural framework of the virtual spider-like embryo

We modeled a spider-like embryo as a yolk-containing elastic ball with a surface occupied by a single layer of packed epithelial cells. We assumed that the shape of each epithelial cell was represented by the two-dimensional shape of its apical surface, which was expressed as a polygon. Epithelial tissue was modeled as a collective polygon defined by connections of vertices. The positions of vertices are the main dynamic variables in the cell vertex model (Honda, 1983). To model the embryo, we used object-oriented computer programming (C++) to construct the vertex, cell, and tissue using a hierarchical relationship. The coordinates of each vertex *i* denote $\vec{r_{i}}$ = (*x*_*i*_, *y*_*i*_, *z*_*i*_) with respect to the center of the spherical embryo. Based on the assumption that the tissue contraction force is balanced with the repulsive force derived from the egg contents, we introduced a restoring force with a spring that constrained the vertices to the surface of the sphere:

$$\frac{d\vec{r_{i}}}{dt}={\vec{F}}_{constraint}=-k_{i}\left(R_{i}-R_{0i}\right)\frac{\vec{r_{i}}}{\left|\vec{r_{i}}\right|}.$$ (Eq. 1)

The constraint force to the spherical surface ${\vec{F}}_{constra\mathrm{int}}$ is exerted on vertex *i*, while the length of sphere $R_{i}=\left|\vec{r_{i}}\right|$ approaches the preferred length of *R*_*0i*_ (*R*_*0i*_ = 270 μm radius for the spider *P. tepidariorum* embryo). The parameter *k*_*i*_ = 30 for all vertices.

### 2.2 Formulating the cell dynamics

The cell vertex model is useful for simulating the mechanical deformation of cells in tissues based on the forces acting on each cell, where the cell configurations are described as polygons whose vertices form cell junctions when subjected to mechanical forces (Farhadifar et al., 2007; Fletcher et al., 2014). Cells change their shape based on force balance. The model is represented by ordinary differential equations of the position vector of each vertex:

$$\frac{d\vec{r_{i}}}{dt}={\vec{F}}_{area\;elasticity}+{\vec{F}}_{adhesion}+{\vec{F}}_{contraction}=-\frac{dE}{d\vec{r_{i}}},$$ (Eq. 2)

$$E=\underset{n}{\sum }\frac{1}{2}\alpha_{n}{\left(A_{n}-A_{0n}\right)}^{2}+\underset{i,j}{\sum }\beta_{ij}\left(t\right)L_{ij}+\underset{n}{\sum }\frac{1}{2}\gamma_{n}{\left(P_{n}-P_{0n}\right)}^{2}.$$ (Eq. 3)

The area elasticity ${\vec{F}}_{area\;elasticity}$ is exerted on vertex *i* by the cell face *n,* to which vertex *i* belongs, while the area of cell *A*_*n*_ approaches the preferred area of *A*_*0*_. The tension at the cell–cell adhesion interface ${\vec{F}}_{adhesion}$ is exerted on vertex *i* by the connecting edges between vertices *i* and *j*, where the cell adhesion increases as the edge length between vertices *i* and *j* (*L*_*ij*_) increases depending on the cell adhesion parameter $\beta_{ij}\left(t\right)$ . The magnitude of cell adhesion parameter $\beta_{ij}\left(t\right)$ changes over time, owing to implementation of interactive cell polarity directions of cells facing each cell edge, described in a later paragraph (Eq. 7 in Section 2.3). The cell contraction ${\vec{F}}_{contraction}$ is exerted on vertex *i* by the perimeter of cell *P*_*n*_, while ${\vec{F}}_{contraction}$ increases to minimize the difference between the perimeter *P*_*n*_ and the preferred perimeter *P*_*0n*_. Taken together, the following defines the differential changes in the position of each vertex, including the constraint force:

$$\frac{d\vec{r_{i}}}{dt}={\vec{F}}_{constraint}+{\vec{F}}_{area\;elasticity}+{\vec{F}}_{adhesion}+{\vec{F}}_{contraction}.$$ (Eq. 4)

We integrated the cell vertex model numerically using the Euler method and confirmed that the results were not greatly influenced by the choice of the temporal discretization size *dt*.

### 2.3 Cell differentiation and cell polarity

We set two conditions in the virtual embryo, which were intended to mimic the contraction (germ disc formation) and the embryo elongation (germ band formation) phases. The multicell was grouped into two cell types that reflected cell differentiation: embryonic and abembryonic for germ disc formation or embryonic and extraembryonic for germ band formation, where cell mechanical parameters were set to be different depending on each cell type.

For the embryo elongation phase (germ band formation), planar cell polarity in an embryonic cell *n* denotes a time-development vector ${\vec{g}}_{n}\left(t\right)$ (*n* = 1 … N, where N is the number of embryonic cells). We assume that the change in the polarity direction of cell *n* is calculated by the difference between the polarity vectors of its neighboring cells *m* (*m* = 1, … , *M*_*n*_) and its own polarity vector as follows:

$$\Delta {\vec{g}}_{n}=\frac{\sum_{m=1}^{M_{n}}\left({\vec{g}}_{m}-{\vec{g}}_{n}\right)}{M_{n}}.$$ (Eq. 5)

Then, the cell polarity in the next time step direction ${\vec{g}}_{n}\left(t+1\right)$ is determined by adding the difference of the cell polarity to the current cell polarity ${\vec{g}}_{n}\left(t\right)$ :

$${\vec{g}}_{n}\left(t+1\right)={\vec{g}}_{n}\left(t\right)+k_{fd}\Delta {\vec{g}}_{n}.$$ (Eq. 6)

The magnitude of *k*_*fd*_ represents the feedback strength. The direction of cell polarity is in parallel with the plane determined by the cell vertices, the position of which is kept close to the surface of the sphere owing to the spring term in the motion equation (Eq. 4). In the virtual embryo with a large number of cells (approximately 3,000 or more), the cell and its neighboring cells are aligned in nearly an identical plane close to the spherical surface. This situation makes the collective vector, calculated from the main and surrounding cells in Eq. 5, stay nearly tangential to the spherical surface over time. The cell adhesion parameters of each cell edge underwent time-dependent changes according to the cell polarity direction. We adopted a similar scheme for coupling the cell adhesion and the cell polarity to that described in a previous study (Sato et al., 2015):

$$\beta_{ij}\left(t\right)=\frac{\beta_{0ij}}{2}\left(1+G\cos\left(\theta_{{\vec{g}}_{n}}-\theta_{ij}\right)\right)+\frac{\beta_{0ij}}{2}\left(1+G\cos\left(\theta_{{\vec{g}}_{m}}-\theta_{ij}\right)\right).$$ (Eq. 7)

The adhesion parameters of each cell edge $\beta_{ij}\left(t\right)$ were the summation of the adhesion parameters of 2 cells *n* and *m* facing their cell edge. The adhesion parameters of each cell *n* or *m* were determined by the difference between the direction of cell polarity $\theta_{{\vec{g}}_{n}}$ or $\theta_{{\vec{g}}_{m}}$ and the direction of cell edge facing these cells $\theta_{ij}$ (−180^o^ $<\theta_{{\vec{g}}_{n}},\;\theta_{{\vec{g}}_{m}},\theta_{ij}\leq$ 180^o^). At the connecting edges between vertices *i* and *j*, the direction of cell edge from *i* to *j* is the opposite direction from *j* to *i* ( $\cos\theta_{ij}=-\cos\theta_{ji}$ ). *G* denotes the strength of the cell polarity, which affects the cell edge adhesion parameter. In germ band formation, initial cell polarities are only set in cells on the rim of the germ disc, and the cell polarities of other cells are set to ${\vec{g}}_{n}\left(t=0\right)=0$ . The initial directions of cell polarity rely on the orientation of the anterior–posterior and dorsal–ventral axes at the germ disc; ${\vec{g}}_{n}\left(t=0\right)=\left(\frac{\sqrt{y_{n}^{2}+z_{n}^{2}}}{R_{0i}}\left(1+\frac{z_{n}}{4R_{0i}}\right),\;\frac{x_{n}}{\sqrt{2}R_{0i}}-\frac{2z_{n}}{R_{0i}},\;\frac{x_{n}}{\sqrt{2}R_{0i}}+\frac{2y_{n}z_{n}}{R_{0i}\sqrt{y_{n}^{2}+z_{n}^{2}}}\right)$ in cells on the rim of the germ disc, where $x_{n}$ , $y_{n}$ , and $z_{n}$ are coordinates of the geometric center of cells.

### 2.4 Mechanical parameter setting

For the contraction phase (germ disc formation), we set the cell mechanical parameters in Eq. 3 using an expansion parameter $\alpha_{n}$ = 1 μm^−2^, an adhesion parameter $\beta_{0ij}$ = 1 μm, and a contraction parameter $\gamma_{n}$ = 2.5 in embryonic cells, and to $\alpha_{n}$ = 1 μm^−2^, $\beta_{0ij}$ = 1 μm, and $\gamma_{n}$ = 0.5 in abembryonic cells. The adhesion parameter of the cell edge between the embryonic and abembryonic cells was $\beta_{0ij}$ = 40 μm.

For the embryo elongation phase (germ band formation), we set the cell mechanical parameters in Eq. 3 using an expansion parameter $\alpha_{n}$ = 1 μm^−2^, an adhesion parameter $\beta_{0ij}$ = 1 μm, and a contraction parameter $\gamma_{n}$ = 0.5 in embryonic cells, and to $\alpha_{n}$ = 0.1 μm^−2^, $\beta_{0ij}$ = 0.1 μm and $\gamma_{n}$ = 0.15 in extraembryonic cells. The adhesion parameter of the cell edge between the embryonic and extraembryonic cells was $\beta_{0ij}$ = 40 μm. The feedback strength of cell polarity *k*_*fd*_ in Eq. 6 was 0.1 in embryonic cells but 0 in extraembryonic cells. The strength of the effect of cell polarity on cell adhesion *G* in Eq. 7 was 10 in embryonic cells but 0 in extraembryonic cells.

### 2.5 Cell division and cell growth

The cell division plane was automatically determined based on the geometric shape of the cell by dividing along the short axis direction of the approximate ellipse for the cell through the geometric center of the cell. The developmental times during germ disc or germ band formation ranged from *t* = 0 (start) to *t* = 1.0 (end). During germ disc formation, the number of embryonic cells increased from *n* = 64 to *n* = 1500 by cell division (where cell cycles follow a normal distribution with mean *t* = 0.2 and variance 0.05). During germ band formation, cell numbers increased again from *n* = 1500 to *n* = 6000 by cell division (where cell cycles follow a normal distribution with mean *t* = 0.5 and variance 0.1). The variation in the timing of the first cell divisions followed a uniform distribution and a range of a quarter of the time (*t* = 0.25) it took for germ disc or germ band formation.

The initial ideal cell area in Eq. 3 was set to $A_{0n}=6,400\;\mu m^{2}$ for all cells in germ disc formation and $A_{0n}=200\;\mu m^{2}$ for all cells in germ band formation. Embryonic cells have no cell area growth, but $A_{0n}$ splits by one half in each cell division step. The abembryonic cells set in a part of embryonic cells (*n* = 100 at *t* = 0.5) and grow by $\frac{dA_{0n}}{dt}=0.001\times 200$ during germ disc formation, and the extraembryonic cells grow by $\frac{dA_{0n}}{dt}=0.009\times 200$ during germ band formation without cell division. The ideal cell perimeter in Eq. 3 was set to *P*_*0n*_ = 0 μm for all cells during both germ disc formation and germ band formation.

### 2.6 Cell rearrangement and cell extrusion

**FIGURE 2:**
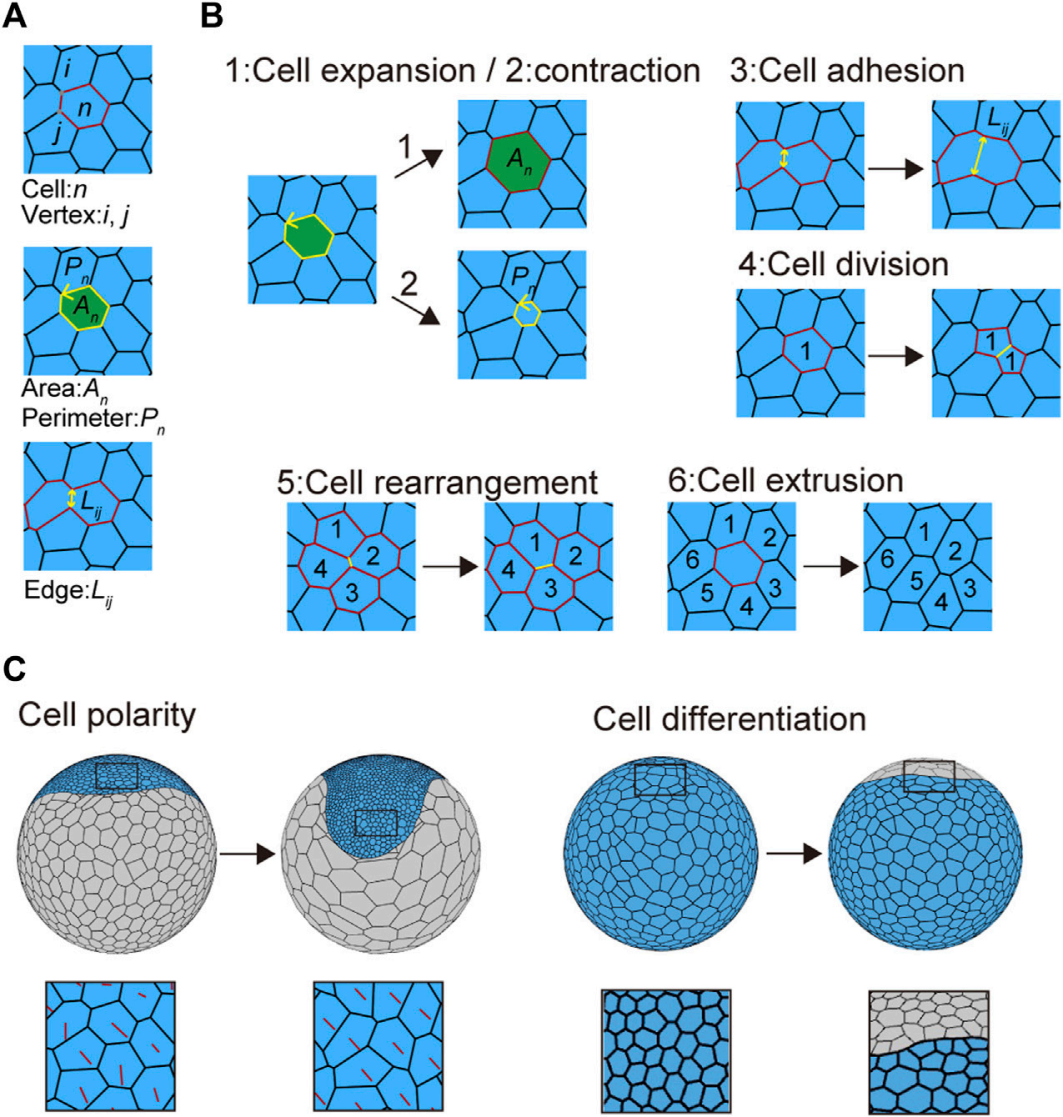
Mechanical cell properties in embryos. **(A)** Virtual cells in the vertex model. The letters *n*, (*i*, *j*), *A*_*n*_, *P*_*n*_, and *L*_*ij*_ in the panels represent the cell number, vertex number, cell area, cell perimeter, and cell edge length, respectively. **(B)** Cell behaviors introduced into the cell vertex model. Three cell mechanics are modeled: 1) cell expansion, 2) cell contraction, and 3) cell adhesion. 1) Cell expansion and 2) cell contraction mainly contribute to increasing or decreasing cell area (*A*_*n*_) and perimeter (*P*_*n*_), whereas 3) cell adhesion mainly contributes to increasing or decreasing cell edge length (*L*_*ij*_; see in Eq. 3 in Methods). Three types of context-dependent cellular events are modeled: 4) cell division, 5) cell rearrangement, and 6) cell extrusion. Numbers in the panels indicate cell positional relationships. Black arrows indicate time advanced. See Supplementary Movie S3 for details. **(C)** Cell polarity (left) and cell differentiation (right) associated with cell shaping. Red lines indicate cell polarity direction (left bottom), while blue and gray cells indicate embryonic and abembryonic cell populations, respectively (right).

Cell rearrangement was implemented through cell neighbor exchange (also called a T1 transition). The T1 transition occurs when the distance between two connected vertices becomes less than a minimum threshold distance (<3 μm), much smaller than a typical edge length (∼10 μm). The connections of vertices are switched as illustrated in Figure 2B(5).

Cell extrusion was implemented by removing the shrunk cell (also called a T2 transition). The T2 transition occurs when the cell area became less than a minimum threshold area (<50 μm^2^) at a triangular cell. The cell was removed, and the geometric center of the removed cell was added to vertices of neighboring cells as a new vertex; cell edges were then connected between the new vertex and its nearest vertices of neighboring cells, as illustrated in Figure 2B(6).

### 2.7 Embedding a reaction and diffusion system

We embedded a reaction and diffusion system in the cell vertex model. Each cell includes concentrations of three types of molecules (*X*_*n*_ ≥ 0, *Y*_*n*_ ≥ 0, and *Z*_*n*_ ≥ 0). These molecules react according to the molecular network in each cell (Eq. 9 and Figure 7C) and diffuse between neighboring cells. The reaction–diffusion equations are written as follows:

$$\begin{array}{l} \frac{dX_{n}}{dt}=R_{x}\left(X_{n},Y_{n},Z_{n}\right)+D_{x}\frac{\partial X^{2}}{\partial^{2}r},\\ \frac{dY_{n}}{dt}=R_{Y}\left(X_{n},Y_{n},Z_{n}\right)+D_{Y}\frac{\partial Y^{2}}{\partial^{2}r},\\ \frac{dZ_{n}}{dt}=R_{Z}\left(X_{n},Y_{n},Z_{n}\right)+D_{Z}\frac{\partial Z^{2}}{\partial^{2}r}. \end{array}$$ (Eq. 8)

In the simulation, we set the reaction equation of the molecular network as an example of wave traveling and subsequent wave splitting by:

$$\begin{array}{l} R_{X}\left(X_{n},Y_{n},Z_{n}\right)=A_{X}X_{0}-B_{X}Y_{n},\\ R_{Y}\left(X_{n},Y_{n},Z_{n}\right)=A_{Y}Y_{n}-{\left(Y_{n}-Y_{0}\right)}^{3}-B_{Y}X_{n}+C_{y}Z_{n},\\ R_{Z}\left(X_{n},Y_{n},Z_{n}\right)=A_{Z}X_{n}-B_{Z}Y_{n}-C_{Z}Z_{n}. \end{array}$$ (Eq. 9)

The parameters were *A*_*X*_ = 1.2, *B*_*X*_ = 1.0, *A*_*Y*_ = 0.2, *B*_*Y*_ = 1.0, *C*_*Y*_ = 1.2, *A*_*Z*_ = 1.0, *B*_*Z*_ = 1.0, *C*_*Z*_ = 0.5, and *Y*_*0*_ = 1.0.

During germ band formation, the initial pattern of these molecules was $X_{0}=1.0$ on the rim of the embryo. Under these parameter settings, the gene expression waves exhibited oscillations and wave-splitting patterns (Figure 7B). The distance related to diffusion $\partial^{2}r$ in Eq. 8 was calculated by the square differential distance ∂r between the geometric center of cell *n* and the geometric centers of its neighboring cells. The diffusion coefficients were *D*_*X*_ = 20, *D*_*Y*_ = 5, and *D*_*Z*_ = 0; thus, *X*_*n*_ and *Y*_*n*_ are diffused, but *Z*_*n*_ is not diffused.

### 2.8 Code for the spherical-surfaced vertex model

The spherical-surfaced vertex model code was written in C++. The code is available at GitHub with the following URL:

https://github.com/Motohiro-Fujiwara/spherical_vertexmodel_spider.git.

Operation checks were made using operating systems for Mac and Linux.

### 2.9 Spiders and culture conditions

Animal experiments were reviewed and approved by the Institutional Animal Care and Use Committee of the JT Biohistory Research Hall (No. 2020–1). Laboratory stocks of the spider *Parasteatoda tepidariorum* (syn. *Achaearanea tepidariorum*) were maintained at 25°C in a 16 h light/8 h dark cycle. The developmental stages have been described previously (Akiyama-Oda and Oda, 2003).

### 2.10 Live imaging and image processing

The developmental stages of the spider embryos were assessed at the start of stage 2 (10 h after egg laying: AEL), stage 5 (30 h AEL), and stage 6 (40 h AEL). Live embryos were dechorionated with 100% commercial bleach, transferred onto heptane-extracted glue in the intended region of a specially designed glass slide, and covered with halocarbon oil 700 (Sigma-Aldrich H8898). Glass capillaries (Drummond 2-000-075) were pulled using a puller (PN-3; Narishige) to make injection needles. Vital fluorescent dyes SPY505-DNA and SPY555-actin (Spiro Chrome), each of which dissolved in DMSO, were mixed at a 1:1 ratio and microinjected into the perivitelline space of the embryos using a needle. The embryos were examined using a Zeiss Axio Zoom.V16 equipped with a digital camera Zeiss Axiocam 506, 2 or 3 hours after injection. Optical sections were made at 4-μm thickness for DNA images (Figure 1A), and 2-μm optical sections from the same embryos were collected for actin and DNA images using an ApoTome3 sectioning unit (Figures 1B and C). Time-lapse images of 2-μm optical sections for DNA and actin were taken at 5 min intervals for 2 h (from 53 to 55 h AEL) using the ApoTome3 sectioning unit (Figures 1D and E). The observed embryos were further examined if embryogenesis had proceeded.

The acquired Z-series images at each time point were processed with ImageJ (FIJI) extended depth of field plugin to generate in-focus single images. Images taken without ApoTome3 were processed using a real wavelet transform with the parameter settings spline order 3 and number of scales 11, while images taken with ApoTome3 used a complex wavelet transform with the parameter settings filter length 6 and number of scales 3. The resultant images were adjusted for brightness and contrast using ImageJ, and the DNA and actin images were merged (Figures 1C–E).

Images of 5-μm optical sections were taken using the same microscope with bright light at 5 min intervals for 3 days. The images at each time point were processed with the ImageJ plugin for the following method and settings: real wavelet, spline order 3, and number of scales 7. The resulting images are compiled to generate Supplementary Movie S1.

## 3 Results and discussion

### 3.1 Observation of the multicellular architecture of the developing spider embryo

We observed the multicellular architecture development of the spider *P. tepidariorum* embryo from early to mid-stages using vital fluorescent dye-labeled DNA and F-actin (Figures 1A–C and Supplementary Movie S1). The spherically symmetric blastoderm forms around 10 h AEL, with approximately 64 cells evenly distributed on the surface of the egg. Two cell populations appeared approximately 15 h AEL, manifesting an axis of radial symmetry in the embryo. One cell population showed stronger concentrations of cortical F-actin and an increasingly denser distribution of cells, whereas the other showed little cortical F-actin and an increasingly sparse distribution of cells. Most of the former cell population participated in the formation of a germ disc, which was a single layer of more than 1,000 epithelial cells that mostly contributed to the ectoderm. Further separation of the germ disc epithelial cell population occurs in the following stages. A small cluster of cells called cumulus mesenchymal (CM) cells was internalized at the center of the germ disc, followed by symmetry-breaking migration along the basal side of the germ-disc epithelium that reached the rim of the germ disc (Akiyama-Oda and Oda, 2010). In a peripheral region of the germ disc where the CM cells arrived, they induced the differentiation of extraembryonic cells (Akiyama-Oda and Oda, 2006), which had progressively larger apical surface areas and less prominent cortical F-actin. Simultaneously, the remaining ectoderm underwent remodeling to form a segmented germ band (Hemmi et al., 2018). During this remodeling process, mediolaterally oriented cell intercalations and variously oriented cell divisions were observed (Figures 1D and E and Supplementary Movie S2), which was consistent with previous reports (Hemmi et al., 2018). These cell dynamics promote tissue deformation to shape the whole embryo.

### 3.2 Constructing a cell vertex model of the spherical epithelial multicell that corresponds to the hierarchical structure of the embryo

The characteristic shape of a developing spider embryo was composed of outer epithelial tissues, which were deformed by epithelial cell dynamics on the spherical surface during embryogenesis (Figure 1A; Akiyama-Oda and Oda, 2010). To reproduce this embryonic shaping process, we constructed a spherical-surfaced vertex model as a virtual multicellular platform (Figure 1F; Honda, 1983; Farhadifar et al., 2007; Fletcher et al., 2014). One advantage of the spherical-surfaced vertex model that distinguishes it from an ordinary 2D sheet vertex model is that it adopts a closed structure system and does not require cell-free boundaries of tissues (Figure 1F). In the constructed vertex model, each polygon represents the apical area of an individual epithelial cell on the embryo surface, and the collective polygons represent the multicellular architecture of the outer epithelial tissue (Figure 1F). We assumed that a whole embryo consisted of one or more tissue types, and that each tissue type consisted of homogeneous cells with certain cell properties. This hierarchical framework of an actual embryo (Figure 1F) was retained in our modeling scheme using object-oriented programming in C++. We also assumed a spherical radius constraint on each vertex to maintain an elastic spherical shape (Eq. 1), which represented the epithelial cell populations attached to inner spherical structures such as the yolk. The connections of polygon edges at each vertex were flexible, and the vertices could increase or decrease in number. These geometric properties allowed the vertex model to express cellular dynamics, such as cell division and cell–cell interactions (Figure 1F and Supplementary Movie S1), as seen in actual embryonic development (Figures 1D and E).

To determine the mechanical properties of individual cell units, we assumed the presence of three sources of potential energy: 1) area elasticity, 2) perimeter contraction, and 3) line adhesion (Figures 2A and B and Supplementary Movie S3). These were expressed in the motion equations for each vertex (Eq. 3; Farhadifar et al., 2007; Fletcher et al., 2014). In addition to these mechanical properties, three types of context-dependent cellular events were included in the model: 4) cell division, 5) cell rearrangement, and 6) cell extrusion, by setting cell cycle periods and transition thresholds (Figure 2B, Supplementary Movie S3 and Methods Section 2.5 and Section 2.6; Farhadifar et al., 2007; Fletcher et al., 2014). Cell shapes were determined by regulating these six cell properties. Changing some of these parameters led to various phenotypic consequences. For example, when it was difficult for cell rearrangements to occur, the embryonic cells showed aberrant shapes (Supplementary Figure S1A).

To reflect global polarity that forms in the cell population along the future anterior–posterior (A-P) and dorsal–ventral (D-V) axes at earlier stages of embryonic development (Akiyama-Oda and Oda, 2006; Akiyama-Oda and Oda, 2020), we assumed that embryonic cells develop a planar cell polarity (Figure 2C). This polarity parameter, which was included in the adhesion term of the vertex motion equation, was initially set along the primary body axes (A-P and D-V axes) but changed over time in a self-determining mode depending on the neighboring cells (Figure 2C; Eq. 6). The cell polarity orientation determined the anisotropic adhesion at the cell edges (Eq. 7). We also introduced cell differentiation by defining two cell types with distinct mechanical properties, cell size characteristics, and cell division frequencies (Figure 2C). However, the cell division plane was automatically determined based on the geometric shape of the cell to divide the cell along the short axis through its geometric center. Given the two cell-type populations with differing cell mechanical properties, the spherical vertex model could represent various embryo shapes, including disc (germ disc) and band (germ band) shapes.

### 3.3 Germ disc formation is achieved by defining two cell populations with distinct mechanical properties

**FIGURE 3:**
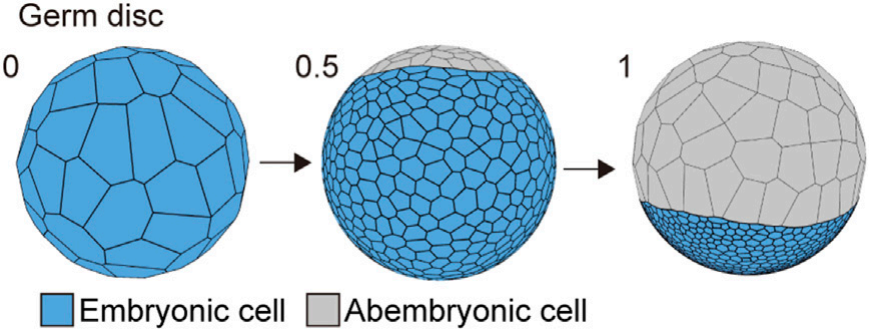
Spherical to germ disc embryo shaping in *P. tepidariorum.* The embryo size was 570 μm diameter, and the cell number ranged from 64 (*t* = 0) to 1,500 (*t* = 1) epithelial cells. When the cell number reached approximately 1,000 cells by cell division (*t* = 0.5), the cells differentiated into two typed-cell populations that were embryonic (blue) and abembryonic (gray) cells.

The first morphogenetic process following the formation of a spherically symmetric blastoderm in the *P. tepidariorum* embryo was the formation of a germ disc (Akiyama-Oda and Oda, 2003; Pechmann, 2016). To mimic this morphogenetic process, we used the cell differentiation for two types of cell populations with distinct mechanical properties when the cell number increased from 64 to 1,000 in the spherical embryo (Figure 3). These two cell populations corresponded to abembryonic and embryonic cells that could form a germ disc. The embryo size (570 μm diameter), cell number, and mechanical parameters for each cell population (differentiation to 900 embryonic cells and 100 abembryonic cells; Methods) were adjusted to achieve a similar morphogenetic process that formed a germ disc (Figure 3; Kanayama et al., 2010). Consistent with the F-actin observation (Figures 1B and C), the embryonic cell population was given a greater adhesion tension and cell contraction strength, while the abembryonic cell population was given a smaller adhesion tension strength. The abembryonic cell population shifted to a non-proliferative state upon differentiation. The adhesion tensions on the cell edges between embryonic and abembryonic cell populations were higher than the adhesion tensions between homogeneous cell populations, which was a requirement for developing a smooth boundary between the two cell populations (Supplementary Figure S1B). These cell mechanics successfully mimicked germ disc formation independent of cell polarity and the emerging axis of radial symmetry. Given that these two cell populations exhibited distinct mechanical properties with appropriate adhesion tension and contraction, a disc-shaped tissue could develop in the spherical-surfaced vertex model.

### 3.4 Germ band formation is achieved by setting two distinct cell types and global polarity

**FIGURE 4:**
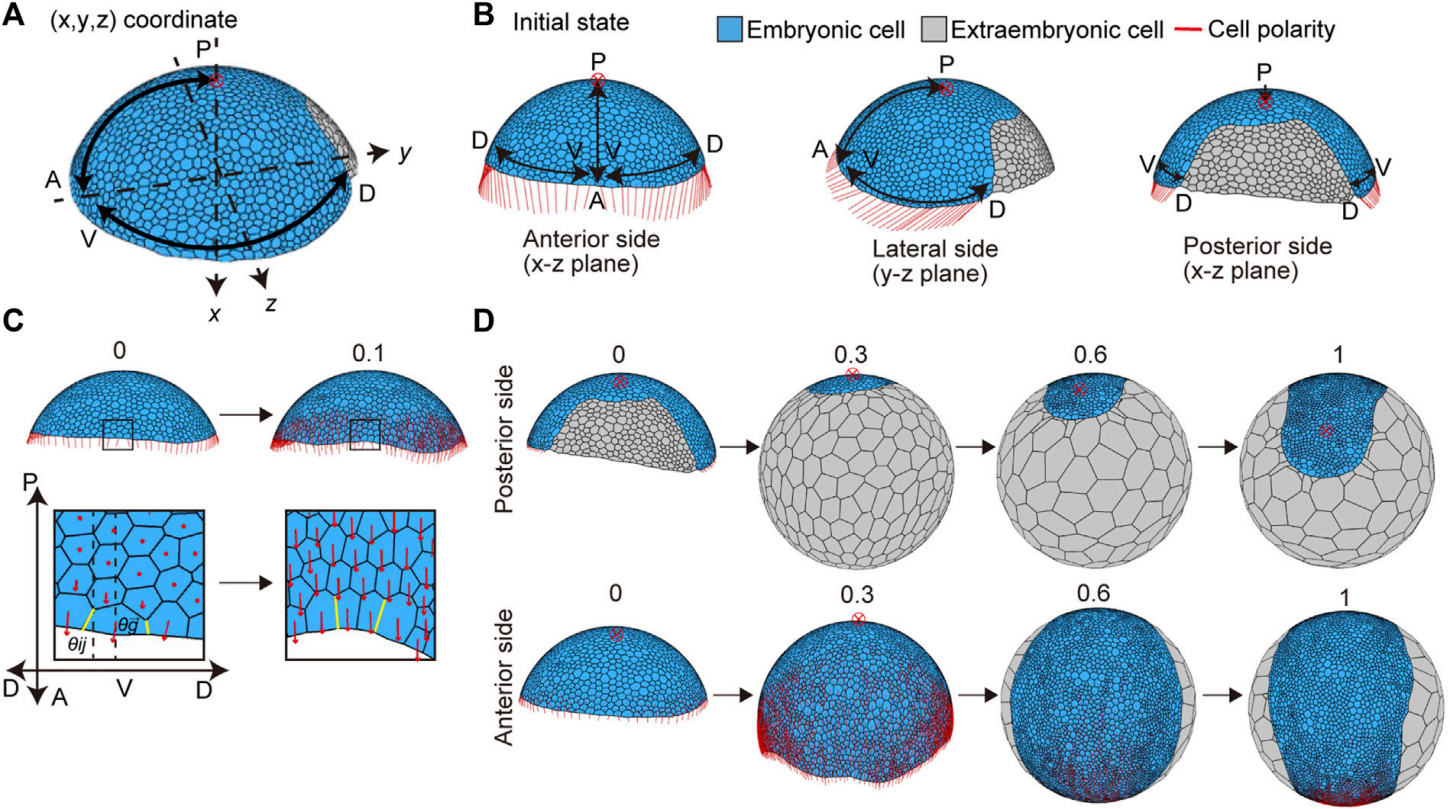
Initial settings for cell polarity and cell differentiation, and the deformation from the germ disc to germ band *in silico*. **(A)** Direction of two body axes (anterior–posterior (A-P) and dorsal–ventral (D-V) axes) defined in the germ disc. The A-P axis is on the x-y plane, and the D-V axis is on the y-z plane in the Cartesian coordinate system (x, y, z). The red cross with a circle indicates the position of a posterior pole in **(A,B,D)**. Embryonic and extraembryonic cells are depicted in blue and gray, respectively in **(A,B,D)**. **(B)** Initial setting for cell polarity, viewed from three different sides of the embryo: the anterior side (left), lateral side (center), and dorsal side (right). Initial cell polarities were only set in embryonic cells located at the rim of the embryo (red line). **(C)** Initial cell polarity defined by the initial direction of the A-P and D-V axes (*t* = 0, left), and changes in the cell polarity over development are shown (*t* = 0.1, right). The boxes in the embryo are magnified in the lower panels. The strengths of cell adhesion tension change depending on different angles between cell edge orientation (yellow line, $\theta_{ij}$ ) and the cell polarity direction ( $\theta_{{\vec{g}}_{n}}$ ; see in Methods). **(D)** Model simulation over time. Initial cell polarities were set as shown in **(B)**. Numbers on each panel represent the developmental time. The virtual disc-like cell assembly (blue) was deformed into a band-like shape, similar to that observed in *P. tepidariorum* embryos.

The *P. tepidariorum* germ disc stage embryo undergoes a dynamic transition process to form a germ band immediately after the signal-sending CM cells arrive at the rim of the germ disc (Akiyama-Oda and Oda, 2006; Hemmi et al., 2018). To mimic this process, the mathematical model assumed two initial conditions for the germ disc cell population (Figure 4A). The first condition was the differentiation of an extraembryonic cell population on the future dorsal side of the germ disc, where the signal-sending CM cells had arrived (Figure 4A). These newly differentiated extraembryonic cells were set to have no proliferative activity and distinct mechanical properties and cell-size characteristics from those of the remaining germ disc cells (Methods). The second condition applied planar cell polarity to reflect the mutually orthogonal A-P and D-V axes of the embryo (Figures 4A and B). Oriented cell polarity was initially set only in the germ disc rim cells (Figure 4B). The directions of cell polarity were interactive among the neighboring cells over time (Eq. 7), which allowed for the effects to spread across the tissue and promote collective cell movement and oriented cell intercalation (Figure 4C). The time evolution of the modeled germ disc started under these two initial conditions (Figures 4B,C) and then mimicked the coordinated dynamics of extraembryonic tissue expansion and germ band formation (Figure 4D and Supplementary Movie S4; center top). The virtual germ band elongated along the emerging A-P axis with frequent mediolaterally oriented intercalations of cells (Figure 4D and Supplementary Movie S1), which was observed in the *P. tepidariorum* embryos (Figure 1D). In this model, cell division frequencies were virtually uniform among the germ band cells, suggesting that locally enhanced cell proliferation is not essential for mimicking germ band formation in spider embryos.

**FIGURE 5:**
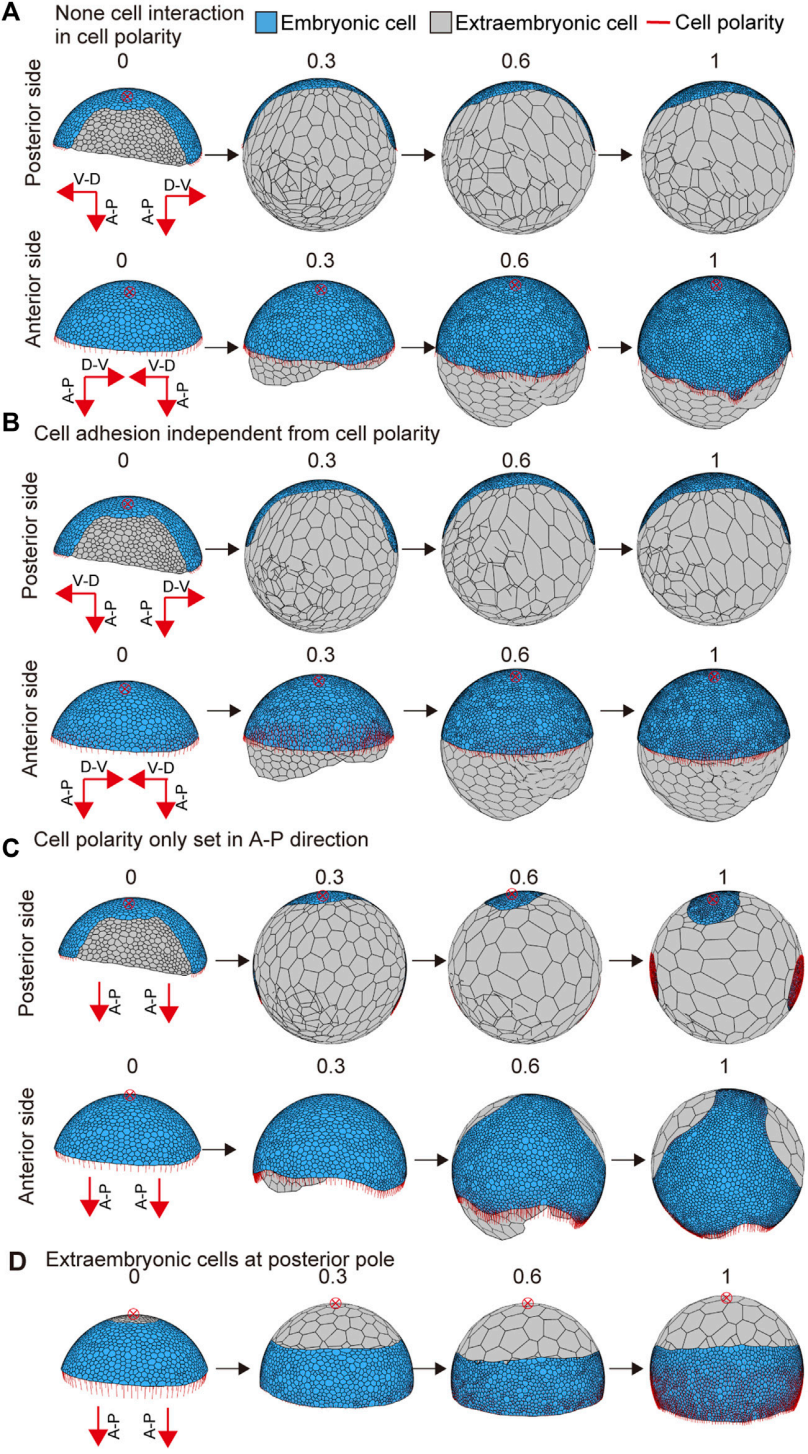
Embryo shape change as affected by parameter values or initial conditions. **(A)** Blocking the interaction between neighboring cells in cell polarity (*k*_*fd*_ = 0 in Eq. 6). The initial conditions for cell polarity are the same as shown in Figure 4B. **(B)** Interrupting the dependency between cell polarity and cell adhesion (*G* = 0 in Eq. 7). The initial conditions for cell polarity are the same as shown in Figure 4B. **(C)** Changing the initial conditions for cell polarity shown in Figure 4B. The development time of embryo shaping when cell polarity was only set in the A-P axis direction. **(D)** Changing the initial conditions for cell differentiation shown in Figure 4B. The development time of embryo shaping when the extraembryonic cells were positioned around the posterior pole of the embryo (red cross with a circle). The red cross with a circle indicates the posterior pole position. Numbers on each panel represent the developmental time (initial state *t* = 0 to after the deformation *t* = 1.0). See Supplementary Movie S4 for details.

To examine the effects of interactive cell polarity and polarity-dependent cell adhesion on embryo shaping, we altered the parameter values to block the respective functions. When the feedback parameter *k*_*fd*_ in Eq. 6 was set to zero to block the interaction between neighboring cells for cell polarity regulation, the embryo was slightly elongated, but no band-like form developed (Figure 5A and Supplementary Movie S4; left bottom). When the polarity dependence parameter for adhesion *G* in Eq. 7 was set to zero, the germ disc did not show any elongation behavior (Figure 5B and Supplementary Movie S4; center bottom). Next, to test the applicability of different initial conditions, we changed the initial cell polarity setting. The polarity of circumferential cells along the rim of the germ disc was set to orient parallel to the A-P axis of the embryo but ignored the global polarity of the D-V axis, with the extraembryonic cell population in the same region (Figure 5C). Under this condition, the simulation of the modeled germ disc resulted in embryo elongation in a direction other than the A-P axis (Figure 5C and Supplementary Movie S4; right top). In another condition, the extraembryonic cell population was initially placed around the center of the germ disc, with the polarity oriented parallel to the A-P axis of the embryo (Figure 5D). The resulting virtual embryo was barrel-shaped (Figure 5D and Supplementary Movie S4; right bottom), mimicking the development of *Pt-patched* knockdown embryos that have signal-sending CM cells that fail to move from the center of the germ disc but still induce differentiation of the extraembryonic cell population (Akiyama-Oda and Oda, 2010). These mathematical simulations using the spherical-surfaced vertex model suggested that spider-like embryonic development can be reproduced with relatively simple model settings. It was also suggested that modifying settings of cell polarity and cell differentiation can cause variation in morphogenetic processes.

**FIGURE 6:**
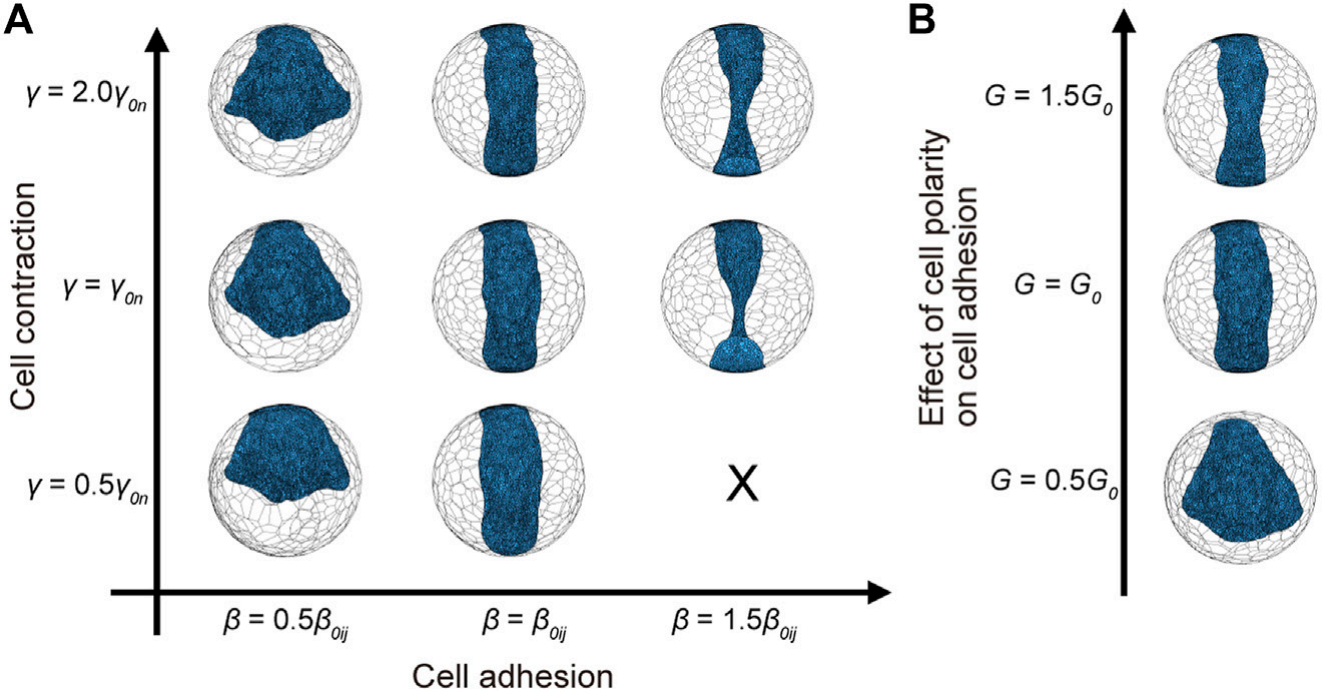
Embryo shape as affected by altering cell mechanical parameters. **(A)** Changing mechanical parameters for cell adhesion $\beta_{0ij}$ in Eq. 7 (horizontal axis) and cell contraction $\gamma_{n}$ in Eq. 3 (vertical axis). Reference parameters in germ band formation as $\beta_{0ij}$ = 1 μm and $\gamma_{0n}$ = 0.5 in embryonic cells. In β = 1.5*β*_0*ij*_ and γ = 0.5*γ*_0*n*_, the numerical calculation was halted before completion (x symbol). In cell adhesion β = $\beta_{0ij}$ , the widths of germ band at the midpoint were 226 μm at $\gamma =0.5\gamma_{0n}$ , 206 μm at $\gamma =\gamma_{0n}$ , and 180 μm at $\gamma =2.0\gamma_{0n}$ . **(B)** Changing the strength of the effect of cell polarity on cell adhesion *G* in Eq. 7. Reference parameters in germ band formation as $G_{0}$ = 10 in embryonic cells. The blue cells represent embryonic cells, and the colorless cells represent extraembryonic cells in **(A,B)**.

Next, we examined the impact of the cell adhesion parameter $\beta_{0ij}$ (in Eq. 7) and the cell contraction parameter $\gamma_{n}$ (in Eq. 3) on the simulation of germ band formation. The parameter β was shifted to 0.5 $\beta_{0ij}$ and to 1.5 $\beta_{0ij}$ , whereas the parameter γ was shifted to 0.5 $\gamma_{0n}$ and to 2.0 $\gamma_{0n}$ (Figure 6A). When the parameter β was 0.5 $\beta_{0ij}$ , the elongation of the forming germ band did not fully occur regardless of the value of the parameter γ (Figure 6A). Conversely, when the parameter β was 1.5 $\beta_{0ij}$ , the formation and elongation of the germ band progressed, but too rapidly, resulting in a germ band being abnormally narrow at the midpoint (Figure 6A). Additionally, we also altered the strength of the effect of cell polarity on cell adhesion *G* (in Eq. 7). When the parameter G was 0.5 $G_{0}$ , the elongation of the forming germ band did not fully occur (Figure 6B). Conversely, when the parameter G was 1.5 $G_{0}$ , the formation and elongation of the germ band progressed, resulting in a germ band being long and narrow (Figure 6B). Taken together, changing the parameters of cell adhesion and cell contraction deformed the germ band outlines, and the strength of cell adhesion was responsible for embryo elongation.

### 3.5 Embedding a genetic network in the spherical-surfaced vertex model

**FIGURE 7:**
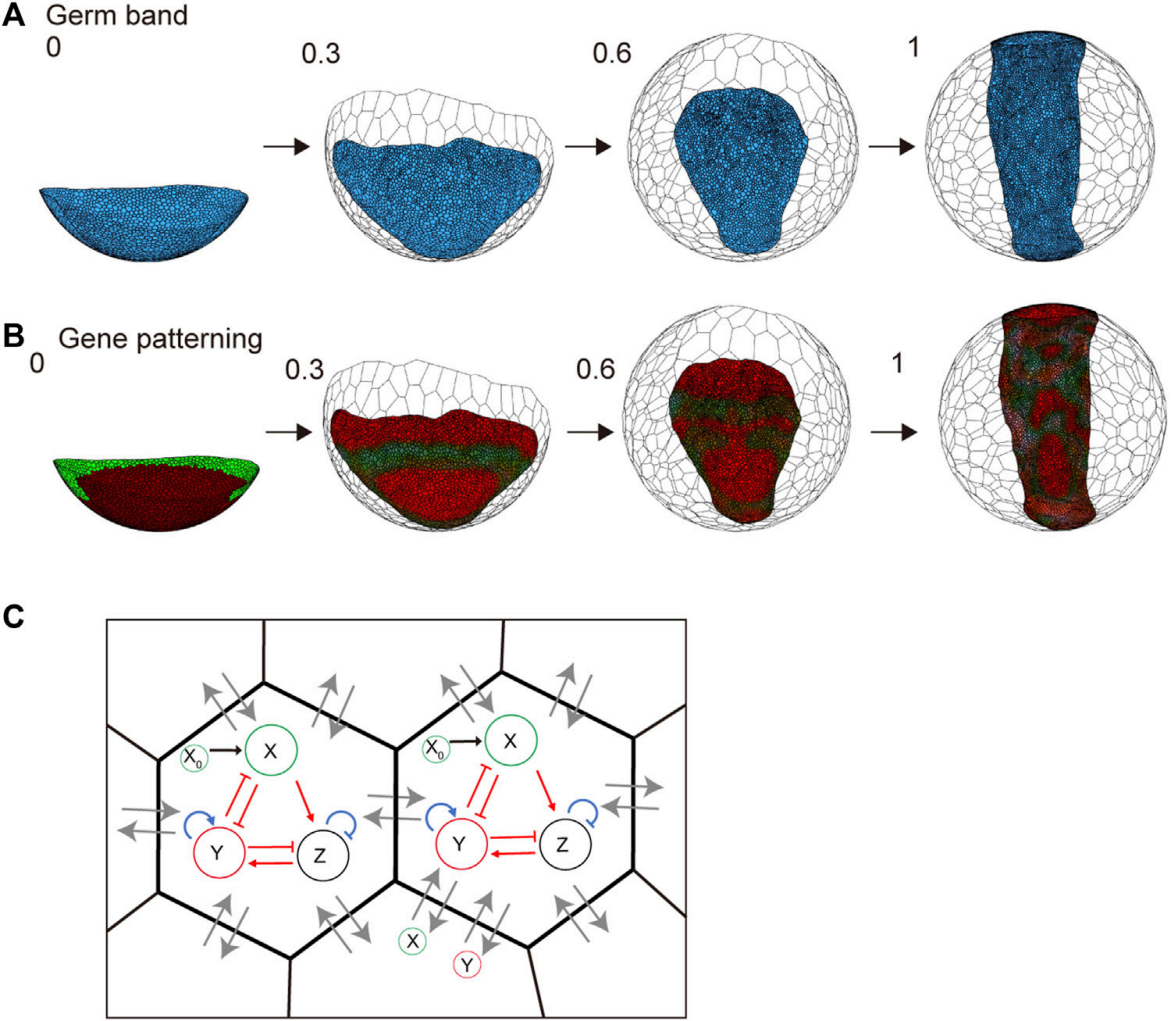
Embryo shaping and gene patterning during germ disc development. **(A)** Body axis formation of the germ band in *P. tepidariorum*. The embryo size is 570 μm diameter, composed of 1,500 (*t* = 0) to 6,000 (*t* = 1.0) epithelial cells. The settings for each cell parameter are described in the Methods section. The blue colored cells represent embryonic cells, and the colorless cells represent extraembryonic cells. **(B)** Gene expression patterning during embryo shaping. The collars indicate the molecules *X* (green) and *Y* (red) defined in **(C)** and Methods section. Numbers on each panel represent the developmental time (initial state *t* = 0 to after the deformation *t* = 1.0). See Supplementary Movie S5 for details. **(C)** Gene expression network in a cell and diffusion between neighboring cells. Red arrows indicate the regulations between two molecules, while blue arrows indicate self-regulation. Black arrows indicate the input from the initial pattern of *X* = *X*_*0*_ (**(B)** left). Lines with arrowheads indicate promotion; those with end bars indicate inhibition. Gray arrows indicate molecular diffusion (*X* and *Y*) between neighboring cells.

In spider embryogenesis, gene expression patterning occurs simultaneously with embryonic shaping (Hemmi et al., 2018). One of the most important goals worth pursuing when using the spherical-surfaced vertex model is to reconstruct the various patterning processes that are controlled by different genetic networks in the field of virtual cells undergoing active rearrangement. Hence, we embedded a simple genetic network with three variables that corresponded to gene activities in the individual cells that form the germ band (Figures 7A–C and Methods). We assumed that the protein products of genes were diffusible with different diffusion coefficients like those in ordinary reaction and diffusion systems that generate stripes, spots, or other patterns (Kondo and Miura, 2010). The embedded genetic network was intended to mimic the wave traveling and splitting of the expression of the spider *hedgehog* (*hh*) homolog (*Pt-hh*), which originates at the rim of the germ disc (Kanayama et al., 2011; Hemmi et al., 2018). The initial gene expression values were set on the embryo edges, as observed in previous studies (Hemmi et al., 2018). Simulations showed that the gene expression wave was followed by splitting when the virtual cellular field underwent germ band formation (Figure 7B and Supplementary Movie S5). However, the integrity of the linear configuration of the transverse gene expression waves was not stably maintained. Cell rearrangements within the plane of the germ band-forming field appeared to cause fluctuations in wave behavior. These cellular dynamics in the patterning field are not usually considered when simulating pattern formation using ordinary reaction and diffusion systems.

In the late *P. tepidariorum* germ disc, differential concentric gene expressions are established along the central–peripheral direction that reflects the future A-P axis under the control of Hedgehog (Hh) signaling. However, the genetic network embedded in the current vertex model does not use this spatial information. This condition may limit the model’s ability to computationally reproduce the pattern-forming behaviors of gene expression waves during germ band formation. In *Drosophila* embryos, regulatory coordination between positional information in a tissue and cell behavior that drives convergent extension has been suggested (Paré et al., 2014). Such a regulatory connection between the emerging rough positional information and cell mechanical parameters should be incorporated to improve our vertex model. Quantitative data on cell position, cell behavior, and gene expression can now be obtained from spider embryos using live imaging, multicolor fluorescent *in situ* hybridization, and single-cell/nuclear transcriptomes (Hemmi et al., 2018; Akiyama-Oda et al., 2022). Analyses of such quantitative data may help us understand the mechanical regulations and genetic networks underlying the pattern-forming processes in the spider embryo.

### 3.6 Future directions for *in silico* evolutionally testing of the body axis formation process in arthropod-like embryos

We have shown that computational simulations using a two-dimensional vertex model modified to operate on a spherical surface can reproduce dynamic cell behaviors that drive the formation of a germ disc and the transition of the germ disc to a germ band similar to those observed in *P. tepidariorum* embryos (Figures 1, 3, 4). In our current model, however, symmetry-breaking steps prior to the two morphogenetic processes are ignored, with the spatial asymmetries given as initial conditions instead. In early spider embryos, there may be localized maternal factors and/or self-determination systems mediated by cell–cell interactions. Regulation of symmetry-breaking CM cell migration is key to achieving continuity during germ disc to germ band development. Although signal-sending CM cells are an internal cell cluster that originates at the polar site, our spherical-surfaced vertex model can be modified to have a signal source that moves below the surface cell layer in response to emerging cues. Previous studies have suggested that these cues are regulated by a genetic network involving Hh signaling in the *P. tepidariorum* embryo (Akiyama-Oda and Oda, 2010). In addition, competence to respond to a signal is an essential property of embryonic cells, and it may be spatially regulated as part of the patterning mechanism. Future models should consider this response as well as a dynamic source of signal.

Hh signaling activity in *P. tepidariorum* embryonic development not only mediates the formation of global polarity but also contributes to the subsequent steps of body axis segmentation (Akiyama-Oda and Oda, 2010; Hemmi et al., 2018; Akiyama-Oda and Oda, 2020). The later activities of Hh signaling, at least in part, may be comparable to those of segment polarity genes in *Drosophila* segmentation. The formation of a spatially periodic striped pattern of *hh* expression is a highly conserved feature in embryonic development in arthropods. Downstream effectors of Hh signaling are involved in regulating the sorting behavior of cells (Larsen et al., 2003), which indicates its potential link to the regulation of cell mechanical properties. This aspect could be incorporated into the cell vertex model. Constructing mathematical models that can reproduce a continuous process by which a spherically symmetric multicellular assembly develops into an arthropod-like segmented body pattern is a long-term goal in future studies.

The germ disc stage, such as in *P. tepidariorum*, is missing in other spider embryos. There are variations in the early embryonic developmental process in many animals, even among spider species (Oda et al., 2020). The virtual multicellular platform proposed in this work is rudimentary but adjustable to different conditions and can be improved. For example, egg shape can be easily modified to test the robustness of a pattern-forming system (Supplementary Figure S1C). Another long-term goal for future studies will be to mathematically test the evolution of arthropod-like embryos.

## 4 Conclusion

We propose a cell vertex model that operates on a spherical surface, where the virtual multicellular system could represent spider-like embryonic development based on the regulation of cell mechanics. The vertex model was implemented with an interactive cell polarity parameter associated with adhesion tension. This implementation allowed for mimicking of the formation of a germ band in spider embryos. This vertex model serves as a virtual multicellular platform to test various spider-like embryonic morphogenetic processes by modifying the parameters and conditions for cell polarity, cell differentiation, and cell mechanical properties. In addition, this multicellular platform has the potential to embed a gene regulatory network that generates waves of gene expression. Further development of the vertex model could contribute to improved reconstruction of arthropod body pattern development and evolution.

## Data Availability

The raw data supporting the conclusion of this article will be made available by the authors, without undue reservation.
