# Supplementary material for: Virtual spherical-shaped multicellular platform for simulating the morphogenetic processes of spider-like body axis formation
Source: Front Cell Dev Biol. 2022 Aug 12;10:932814. doi: 10.3389/fcell.2022.932814 (PMC9411422; doi:10.3389/fcell.2022.932814)
Supplement: Supplementary file 2 [file DataSheet1.pdf]

Supplementary Material

1 Supplementary Material for "Virtual spherical-shaped multicellular platform for simulating the morphogenetic processes of spider-like body axis formation".

1.1 Supplementary Figures

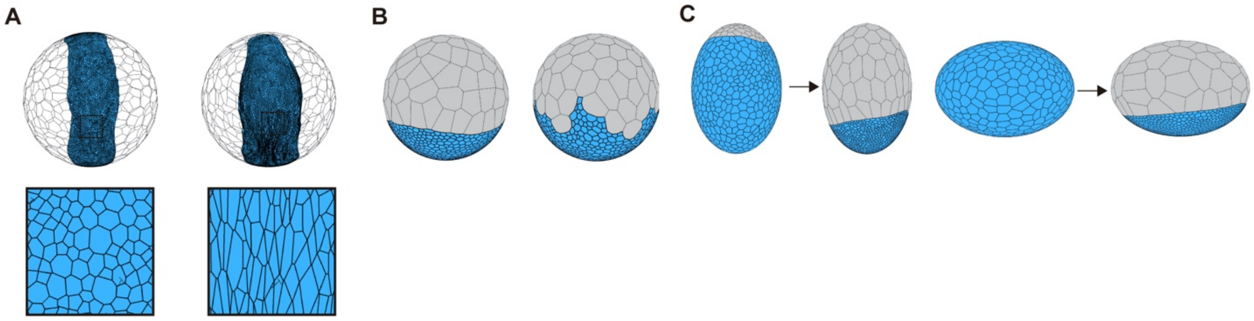

Supplementary Figure S1. Supporting figure for the spherical cell vertex model

(A) Germ band formations with easily occurring cell rearrangement (left; T1 threshold distance < 3 μm) or with no cell rearrangement (right; cell rearrangement was suppressed by prohibiting T1 transition; threshold distance < 0 μm). The lower panels indicate the cell shapes for each condition.

(B) Boundary condition for cell adhesion between two different cell types of multicell (embryonic cells: blue and abembryonic cells: gray). Cell adhesion parameter of the edges between two different cell types β_{0ij}= 40 μm in left panel; β_{0ij}= 1 μm in right panel.

(C) Germ disc formations at changing initial embryo shapes: vertically long ellipsoid (left; vertical diameter = 580 μm and horizontal diameter = 500 μm) and horizontally long ellipsoid (right; vertical diameter = 500 μm and horizontal diameter = 580 μm).

1.2 Supplementary Movies

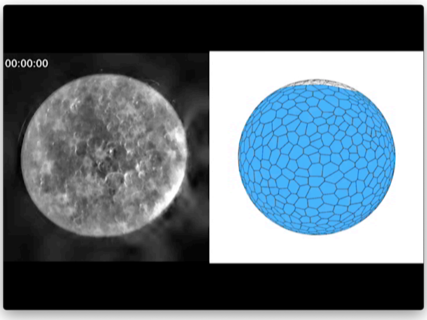

Movie S1. Spider embryogenesis *in vivo* (left) and *in silico* (right).

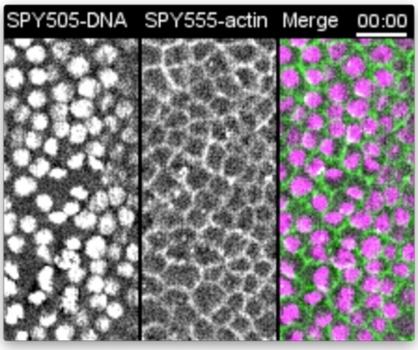

Movie S2. Cell convergent extension for Figure 1D.

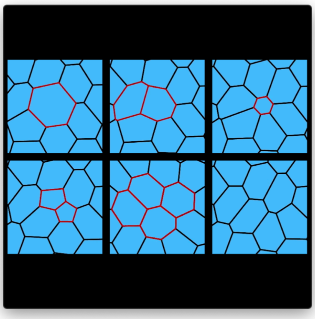

Movie S3. Cell dynamics for each cell behavior shown in Figure 2B. 1) cell expansion (left top), 2) cell contraction (right top), 3) cell adhesion (center top), 4) cell division (left bottom), 5) cell rearrangement (center bottom), and 6) cell extrusion (right bottom).

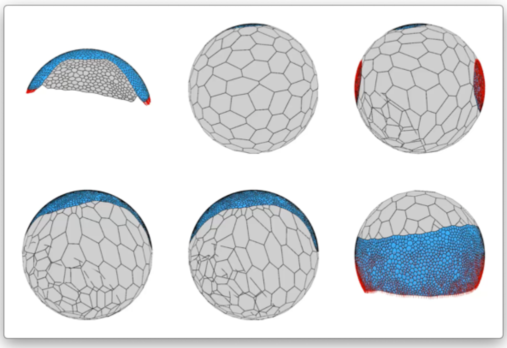

Movie S4. Initial settings in cell polarity (left top), and germ band formation (center top) as for Figure 4. Cell polarity only set in A-P axis (right top), no cell interaction in cell polarity (left bottom), cell adhesion independent from cell polarity (center bottom), and extraembryonic cells at posterior side (right bottom) as for Figure 5.

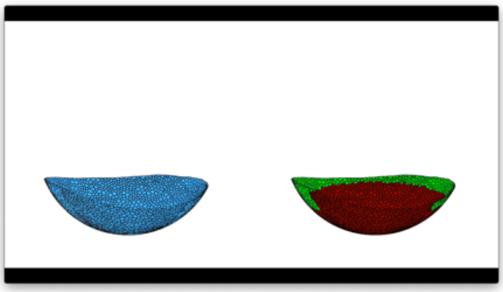

Movie S5. Embryo shaping (left) and gene expression patterning (right) in *P. tepidariorum in silico* for Figure 6.
